# Comparative genomics reveals differences in mobile virulence genes of *Escherichia coli* O103 pathotypes of bovine fecal origin

**DOI:** 10.1371/journal.pone.0191362

**Published:** 2018-02-01

**Authors:** Lance W. Noll, Jay N. Worley, Xun Yang, Pragathi B. Shridhar, Justin B. Ludwig, Xiaorong Shi, Jianfa Bai, Doina Caragea, Jianghong Meng, T. G. Nagaraja

**Affiliations:** 1 Department of Diagnostic Medicine/Pathobiology, Kansas State University, Manhattan, Kansas, United States of America; 2 Joint Institute for Food Safety and Applied Nutrition and Department of Nutrition and Food Science, University of Maryland, College Park, Maryland, United States of America; 3 Veterinary Diagnostic Laboratory, Kansas State University, Manhattan, Kansas, United States of America; 4 Department of Computing and Information Sciences, Kansas State University, Manhattan, Kansas, United States of America; The Pennsylvania State University, UNITED STATES

## Abstract

*Escherichia coli* O103, harbored in the hindgut and shed in the feces of cattle, can be enterohemorrhagic (EHEC), enteropathogenic (EPEC), or putative non-pathotype. The genetic diversity particularly that of virulence gene profiles within O103 serogroup is likely to be broad, considering the wide range in severity of illness. However, virulence descriptions of the *E*. *coli* O103 strains isolated from cattle feces have been primarily limited to major genes, such as Shiga toxin and intimin genes. Less is known about the frequency at which other virulence genes exist or about genes associated with the mobile genetic elements of *E*. *coli* O103 pathotypes. Our objective was to utilize whole genome sequencing (WGS) to identify and compare major and putative virulence genes of EHEC O103 (positive for Shiga toxin gene, *stx*1, and intimin gene, *eae;* n = 43), EPEC O103 (negative for *stx*1 and positive for *eae*; n = 13) and putative non-pathotype O103 strains (negative for *stx* and *eae;* n = 13) isolated from cattle feces. Six strains of EHEC O103 from human clinical cases were also included. All bovine EHEC strains (43/43) and a majority of EPEC (12/13) and putative non-pathotype strains (12/13) were O103:H2 serotype. Both bovine and human EHEC strains had significantly larger average genome sizes (*P* < 0.0001) and were positive for a higher number of adherence and toxin-based virulence genes and genes on mobile elements (prophages, transposable elements, and plasmids) than EPEC or putative non-pathotype strains. The genome size of the three pathotypes positively correlated (R^2^ = 0.7) with the number of genes carried on mobile genetic elements. Bovine strains clustered phylogenetically by pathotypes, which differed in several key virulence genes. The diversity of *E*. *coli* O103 pathotypes shed in cattle feces is likely reflective of the acquisition or loss of virulence genes carried on mobile genetic elements.

## Introduction

Enterohemorrhagic *Escherichia coli* (EHEC) carry one or both phage-encoded Shiga toxin genes (*stx*1 and *stx*2) and the attaching and effacing gene (*eae*), which is harbored in the chromosomal-encoded locus of enterocyte effacement (LEE) pathogenicity island. Among EHEC pathotypes, O157:H7 serotype is most frequently associated with human foodborne illness. However, Centers for Disease Control and Prevention (CDC) rank O103 as the second most common serogroup, next to O26, identified in laboratory confirmed non-O157 EHEC infections in the U.S. [1]. In human EHEC infections, disease outcomes can range from mild to bloody diarrhea (hemorrhagic colitis) to more serious complications, such as hemolytic uremic syndrome (HUS), and even death [2]. Differences in disease-causing potential, particularly the ability to cause serious complications, are attributed to differences in virulence of EHEC strains [3]. In addition to the major virulence factors, which include Shiga toxins and LEE gene-encoded proteins, other virulence attributes, including known putative virulence factors, contribute to the development, progression, and outcome of the disease [4–6]. Enteropathogenic *E*. *coli* (EPEC), including EPEC O103, do not carry *stx* genes; however, they possess *eae* and other virulence genes to cause attaching and effacing lesions that can result in mild to severe diarrhea, or even death, particularly in children [7, 8]. Strains within the EPEC pathotype are further characterized as typical or atypical, depending on presence or absence, respectively, of the EPEC adherence factor (EAF) plasmid [9]. The loss of the *stx* gene(s), a frequently reported event [10, 11], can transform an EHEC into an EPEC pathotype. These major pathotype-defining mobile virulence genes have been well studied, but less is known about how other mobile elements contribute to the overall virulence diversity in O103 serogroup. Some strains of *E*. *coli* O103 carry neither Shiga toxin nor intimin genes, possibly a non-pathotype; even less is known about the virulence profiles of these strains. Cattle have been shown to harbor EHEC, EPEC and putative non-pathotype O103 in the hindgut and shed them in the feces [12]. We hypothesize that the diversity of O103 pathotypes harbored and shed in the feces of cattle is reflective of the loss or acquisition of genes carried on mobile genetic elements.

Whole genome sequencing (WGS) has been used to characterize the virulence gene profiles of EHEC O157 [13], identify phylogenetic relationships between EHEC O157 and non-O157 serotypes [14–18] as well as discover novel virulence determinants [19]. However, differences in virulence gene profiles and phylogenetic relationships of O103 pathotypes of bovine origin are less characterized [20]. Therefore, our objectives were to utilize WGS to identify and compare major and putative virulence genes, particularly genes located on mobile elements, of bovine and human clinical EHEC O103, bovine EPEC O103, and putative non-pathotype O103 strains and analyze phylogenetic relationships among the strains.

## Materials and methods

### Strains

The Institutional Animal Care and Use Committee at Kansas State University approved the research that resulted in the strains that were used in the study. The bovine EHEC strains investigated in this study were isolated from cattle feces from several feedlots in the Midwest region of the US [12, 21, 22]. Sixty-nine bovine O103 strains, previously identified by end-point PCR [23] as positive for *stx*1 (Shiga toxin 1) and *eae* (intimin) (bovine EHEC; n = 43), negative for *stx*1 and positive for *eae* (bovine EPEC; n = 13) and negative for both *stx*1 and *eae* (bovine putative non-pathotype; n = 13) were used in the study. Human clinical O103 strains positive for *stx*1 and *eae* (human EHEC; n = 6) were included in the study for comparison. The strains were cultured onto Tryptone soy agar (TSA; BD Difco, Sparks, MD) slants and shipped overnight in cold storage to the University of Maryland for whole genome sequencing.

### DNA preparation and whole genome sequencing

The O103 strains from TSA slants were streaked onto blood agar (Remel, Lenexa, KS) and then subcultured in tryptone soy broth (BD Difco, Sparks, MD). Bacterial DNA from overnight culture was extracted from each strain using the DNeasy Blood and Tissue Kit with the QIAcube robotic workstation (Qiagen, Germantown, MD). The genomes were sequenced using an Illumina MiSeq platform (Illumina, San Diego, CA) at approximately 37x average coverage. Genomic libraries were constructed using Nextera XT DNA Library Preparation Kit and MiSeq Reagent Kits v2 (500 Cycles) (Illumina, Inc.). *De novo* genome assembly was performed using SPAdes 3.6.0 [24].

### Genomic analysis

Draft genomes were annotated using Rapid Annotation using Subsystem Technology (RAST version 2.0 - http://rast.nmpdr.org/; [25]), a web-based service commonly used for annotation of draft bacterial genomes [26, 27]. RAST applies the Fellowship for Interpretation of Genomes (FIG) subsystem approach to rapidly call and annotate genes, then uses high-throughput comparative analysis and a collection of expertly curated databases to categorize genes, based on the functional role they perform, into subsystems. Average number of genes located on mobile elements (prophages, transposable elements and plasmids), and genes related to virulence, disease and defense were determined, using RAST, for each of the O103 subgroups (bovine EHEC, human EHEC, bovine EPEC and bovine putative non-pathotype). Genomic sequencing data in this study exceeded the minimum criteria for analysis that RAST requires of each genome: at least 10x coverage (using 454 pyrosequencing) and 70% of assembled sequences in contigs > 20,000 base pairs. Serotype identity, virulence and plasmid make-up of the 75 strains were determined using default parameters of Center for Genomic Epidemiology Serotype-Finder 1.1 (https://cge.cbs.dtu.dk/services/SerotypeFinder/) [28],Virulence Finder 1.4 (https://cge.cbs.dtu.dk/services/VirulenceFinder/) [29], and PlasmidFinder 1.3 [30] programs, respectively. Prophage sequences of the 75 strains were determined using Phage Search Tool Enhanced Release (PHASTER; http://phaster.ca/) [31, 32]; intact, and questionable prophage sequences, defined by PHASTER scores of >90 and 70–90, respectively, were included in analysis. The complete genome of EHEC O103:H2 strain 12009 (GenBank accession no. AP010958.1; https://www.ncbi.nlm.nih.gov/nuccore/AP010958.1) and 12009 plasmid pO103 DNA (GenBank accession no. NC_013354.1; https://www.ncbi.nlm.nih.gov/nuccore/NC_013354.1), a classical O103 reference strain of clinical origin used in many O103 genomic studies [14, 33, 34], was tested with Virulence Finder 1.4, ResFinder 2.1, Plasmid Finder 1.3 and PHASTER as a control for comparison. The complete genomes EHEC O157:H7 Sakai (GenBank accession no. BA000007.2; https://www.ncbi.nlm.nih.gov/nuccore/BA000007.2) and EHEC O157:H7 EDL933 (GenBank accession no. CP008957.1; https://www.ncbi.nlm.nih.gov/nuccore/CP008957.1) and their associated plasmids (Sakai plasmid pO157: GenBank accession no. NC_002128.1, https://www.ncbi.nlm.nih.gov/nuccore/NC_002128.1; Sakai plasmid pOSAK1: GenBank accession no. NC_002127.1, https://www.ncbi.nlm.nih.gov/nuccore/NC_002127.1; EDL933 plasmid pO157: GenBank accession no. AF074613.1, https://www.ncbi.nlm.nih.gov/nuccore/AF074613.1) were also tested for comparison. Parsnp v1.2 (http://harvest.readthedocs.io/en/latest/content/parsnp.html) [35] was used for core genome alignment of the 75 strains and subsequent construction of a maximum likelihood tree. For improved visualization, a proportional branch transformation of the output file (.tree) from Parsnp was performed using FigTree 1.4 software (http://tree.bio.ed.ac.uk/software/figtree/) [36] and bootstrap values were reported for each branch. Representative strains, based on clustering patterns observed in the phylogenetic tree, were chosen as input for BLAST Ring Image Generator software (BRIG v0.95 - https://sourceforge.net/projects/brig/) [37]. The BRIG plot displays similarities and differences between the draft genome nucleotide sequence identities of target stains, represented by concentric rings, to the genome identity of a chosen reference strain, identified in the center of the BRIG plot. The complete genome of EHEC O103:H2 strain 12009 was used as a BRIG plot reference. The nucleotide sequence (45,325 bp) of the LEE pathogenicity island (GenBank accession no.: AF071034.1; https://www.ncbi.nlm.nih.gov/nuccore/AF071034.1) of human clinical EHEC O157:H7 EDL933 strain [37] was mapped to the BRIG plot for comparison of LEE between the target strains.

### Statistical analysis

A single factor analysis of variance (ANOVA) test was performed to determine whether average genome size, and average number of extra-chromosomal genes and virulence, disease and defense genes were significantly different among the four subgroups (bovine EHEC, human EHEC, EPEC and putative non-pathotype). If means were significantly different (*P* ≤ 0.01), Tukey adjustment for multiple comparisons was performed, using SAS 9.4 with Proc Glimmix, to test each pairwise comparison for significant differences (*P* ≤ 0.01).

### Nucleotide sequence accession numbers

Draft genome sequences of the 75 *E*. *coli* O103 strains are available in GenBank and their accession numbers are listed in Tables in S1, S2 and S3 Tables.

## Results

Sixty-nine bovine O103 strains, that belonged to three subgroups, EHEC (n = 43), EPEC (n = 13) and putative non-pathotype (n = 13) and six human clinical EHEC O103 strains were included in the study. All bovine EHEC strains (43/43; 100%) and a majority of EPEC (12/13; 92.3%) and putative non-pathotype strains (12/13; 92.3%) were O103:H2 serotype. The two remaining strains of EPEC (1/13) and putative non-pathotype (1/13) were O103:H11 and O103:H16 serotypes, respectively. Four of the six human EHEC strains were O103:H11 and two were O103:H2 serotype.

### RAST subsystem summary

Genome size range of bovine (5.32–5.79 Mb) and human EHEC (5.43–5.77 Mb) subgroups were similar (Table 1). However, both bovine and human EHEC subgroups had significantly larger average genome sizes (*P* ≤ 0.0001) compared to EPEC or putative non-pathotype subgroups. Average genome size was similar between EPEC and putative non-pathotype subgroups. However, one of the bovine EPEC O103:H11 strains (2013-3-492A) had a similar genome size (5.67 Mb) to that of other EHEC strains.

**Table 1:** Average genome size, guanine-cytosine (GC) content, and number of contigs and average number of extra-chromosomal genes, virulence, disease and defense genes and plasmids of enterohemorrhagic (EHEC), enteropathogenic (EPEC) and putative non-pathotype (*stx*/*eae* negative) *Escherichia coli* O103 strains of bovine and human origin.

Genome size and gene categories^†^		Host origin, pathotype and serotype (no. of strains tested)				
	Bovine EHEC	Human EHEC		Bovine EPEC	Bovine putative non-pathotype	
	O103:H2 (n = 43)	O103:H2 (n = 2)	O103:H11 (n = 4)	O103:H2 (n = 12)	O103:H11 (n = 1)	O103:H2 (n = 12)
**Genome size (Mb)**	5.47 (5.32–5.79)	5.45 (5.43–5.46)	5.61 (5.52–5.77)	5.22 (5.16–5.33)	5.67	5.26 (5.21–5.33)
**GC content (%)**	50.58 (50.5–50.6)	50.6 (50.6–50.6)	50.43 (50.4–50.5)	50.54 (50.5–50.6)	50.5	50.44 (50.4–50.5)
**Contigs**	312 (181–398)	350 (339–360)	432 (412–453)	204 (137–268)	406	140 (99–172)
**Virulence, disease and defense**	113 (111–124)	111 (111–111)	112 (109–121)	114 (113–117)	109	115 (115–116)
**Prophages, transposable elements and plasmids**	260 (221–351)	265 (260–270)	273 (256–292)	157(137–213)	289	128 (100–157)
**Plasmids**	2.9 (1–6)	2.5 (2–3)	4 (3–6)	2 (0–4)	5	3 (3–4)

^†^Genome sizes, GC content, contigs, virulence, disease and defense and mobile element (prophages, transposable elements and plasmids) data were determined using Rapid Annotation Using Subsystem Technology (RAST; [25]). Plasmid data was determined using PlasmidFinder 1.3 [30].

Overall, the number of genes in the category of virulence, disease and defense was comparable for all 75 strains tested (Table 1), with no significant differences observed in the mean number of genes among the O103 subgroups. However, the number of genes on mobile elements (prophages, transposable elements, and plasmids) varied considerably among O103 subgroups and among serotypes within subgroups. Strains belonging to bovine and human EHEC subgroups had a significantly higher (*P* ≤ 0.001) number of mobile genes compared to EPEC and putative non-pathotype subgroups. Average number of mobile genes was not significantly different between bovine and human EHEC subgroups or between EPEC and putative non-pathotype subgroups. The bovine EHEC strains possessed the widest range in the number of genes on mobile elements (221–351). Similarly, wide ranges were observed in bovine EPEC strains (137–289 genes) and bovine putative non-pathotype strains (100–157 genes), but not in human EHEC strains (256–292 genes). Mobile gene counts above 300 were only observed in a few bovine EHEC strains (4/43), and one bovine EHEC strain (2014-5-933A) had 351 mobile genes, nearly 60 more than the highest number in strains of the human EHEC subgroup. Furthermore, the one bovine EPEC O103:H11 (strain 2013-3-492A) that had a similar genome size as EHEC pathotype had 289 mobile genes; 76 more mobile genes than the highest number in strains within the EPEC O103:H2 subgroup.

**Fig 1:**
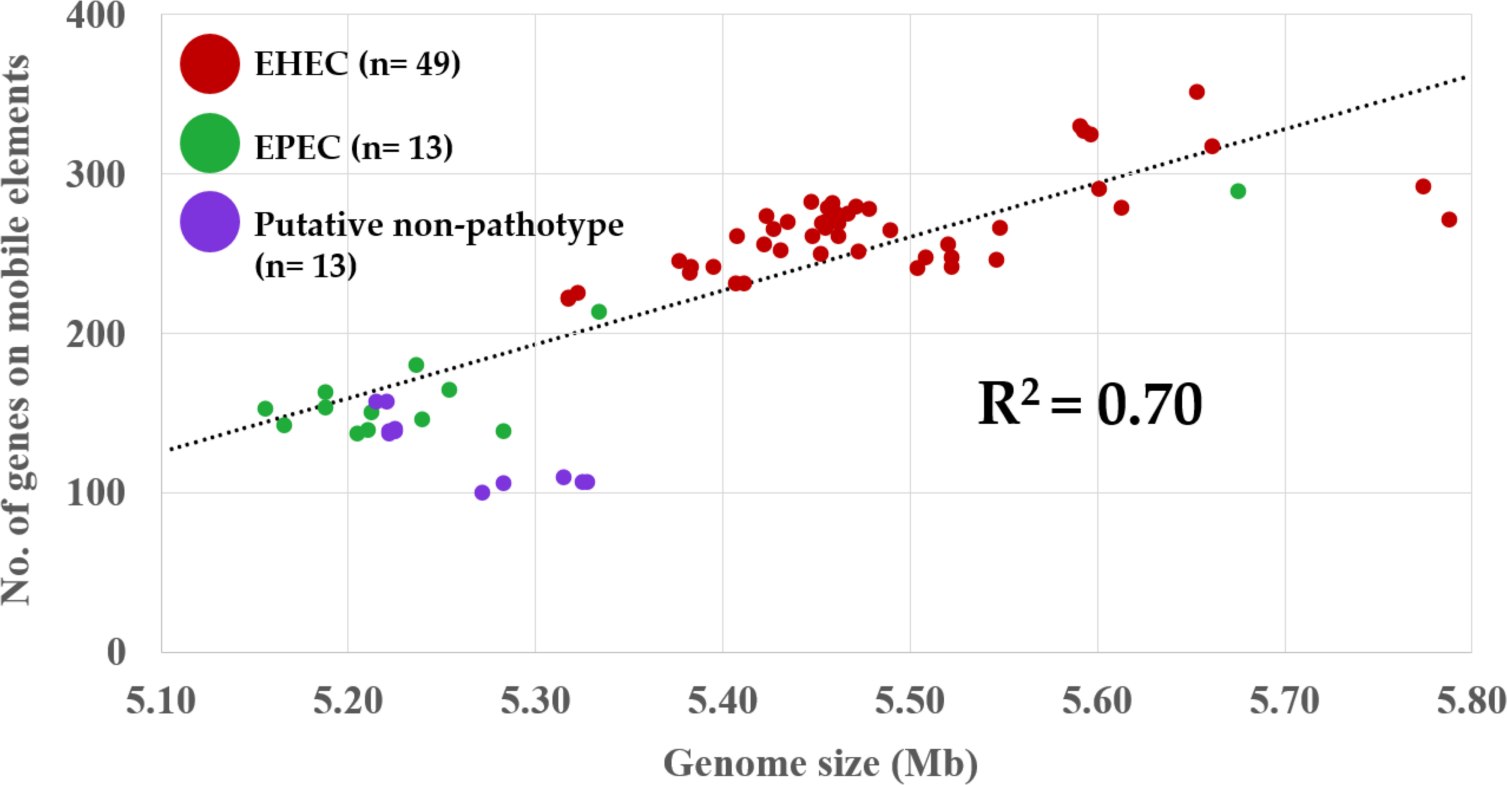
Scatterplot of genome sizes and number of genes on mobile elements^†^ of 75 strains of enterohemorrhagic (EHEC), enteropathogenic (EPEC) and putative non-pathotype (*stx*/*eae* negative) *Escherichia coli* O103. ^†^Genome sizes and number of genes located on mobile elements (prophages, transposable elements and plasmids) were determined using Rapid Annotation Using Subsystem Technology (RAST; [25]).

A strong correlation (R^2^ = 0.70) was observed between genome size vs. number of genes on mobile elements for the 75 strains (Fig 1). The EHEC strains had larger genome size and higher number of genes on mobile elements compared to EPEC and putative non-pathotype strains. The EPEC O103:H11 strain (2013-3-492A) appeared to be an EPEC outlier, with genome size and number of genes on mobile elements closer to those of the EHEC O103 strains (Fig 1).

### Virulence genes

Virulence genes with >90% sequence homology were considered positive in a genome. The complete virulence gene profiles of each genome are shown in tables in S1, S2 and S3 Tables. All EHEC strains were positive for Shiga toxin 1a (*stx*1a) subtype. On average, bovine and human EHEC strains were positive for more virulence genes than EPEC strains; putative non-pathotype strains were negative for all LEE encoded, non-LEE encoded, and pO157 plasmid-encoded genes (Table 2).

**Table 2:** Major chromosomal-, phage-, and plasmid-encoded virulence genes in enterohemorrhagic (EHEC), enteropathogenic (EPEC) and putative non-pathotype (*stx*/*eae* negative) *Escherichia coli* O103 strains of bovine and human origin^†^.

Origin	Protein and gene		Host origin, pathotype and serotype (no. of strains)						
			Bovine EHEC	Human EHEC		Bovine EPEC		Bovine putative non-pathotype	
			O103:H2	O103:H2	O103:H11	O103:H2	O103:H11	O103:H2	O103:H16
			(n = 43)	(n = 2)	(n = 4)	(n = 12)	(n = 1)	(n = 12)	(n = 1)
**Locus of enterocyte effacement (LEE) encoded**	**Intimin**	***eae***	43	2	4	12	1	0	0
	**Translocated intimin receptor**	***tir***	43	2	4	12	1	0	0
	**Type III secretions system**	***espA***	43	2	4	12	1	0	0
	**Secreted protein B**	***espB***	43	2	4	12	1	0	0
	**Type III secretion system**	***espF***	39	2	4	9	1	0	0
**Non-LEE encoded**	**Effector A**	***nleA***	43	2	4	6	1	0	0
	**Effector B**	***nleB***	43	2	4	10	1	0	0
	**Effector C**	***nleC***	23	0	4	0	0	0	0
**Phage-encoded Shiga toxin**	**O157 FLY16, variant a**	***stx*1**	42	2	4	0	0	0	0
	***Shigella dysenteriae* 3818T**	***stx*1**	1	0	0	0	0	0	0
	**Shiga toxin 1, subunit A, variant a**	***stx*1A**	43	2	4	0	0	0	0
	**Shiga toxin 1, subunit B, variant a**	***stx*1B**	43	2	4	0	0	0	0
**Phage-encoded type III secretion effectors**	**Cycle inhibiting factor**	***cif***	43	2	4	0	1	0	0
	**Type III secretion system effector**	***espJ***	38	2	4	0	1	0	0
	**Tir-cytoskeleton coupling protein**	***tccP***	41	2	3	12	0	0	0
**pO157 plasmid-encoded**	**Enterohaemolysin**	***ehxA***	43	2	4	11	1	0	0
	**Extracellular serine protease**	***espP***	37	2	4	11	1	0	0
	**Type II secretion protein**	***etpD***	9	0	0	0	0	0	0
	**Catalase peroxidase**	***katP***	29	2	4	0	1	0	0
	**Toxin B**	***toxB***	1	1	1	0	1	0	0

^†^Virulence genes were determined using Virulence Finder 1.4 [29]).

Among LEE-encoded genes, all EHEC and EPEC strains were positive for *eae*, translocated intimin receptor protein (*tir*), and type III secretion effectors (*esp*A and *esp*B), but a small number of bovine EHEC (4/43) and EPEC O103:H2 strains (3/12) were negative for type III secretion effector gene, *esp*F (Table 2). All EHEC/EPEC O103:H2 and O103:H11 serotypes were positive for *eae*-epsilon and *eae*-beta1 subtypes, respectively. Other phage-encoded type III secretion effector genes (*cif*, *esp*J, and *tcc*P) were present in all human EHEC O103:H2 strains but were present at varying proportions for other EHEC and EPEC O103 subgroups. Non-LEE encoded effectors A (*nle*A) and B (*nle*B) were present in all EHEC strains, in the EPEC O103:H11 strain, but also in a majority of EPEC O103:H2 strains (6/12 for *nle*A and 10/12 for *nle*B). The *nle*C gene, absent in two human EHEC O103:H2 strains, was present in all human EHEC O103:H11 strains (4/4) and also in over half of bovine EHEC O103:H2 strains (23/43; 53.5%).

Among pO157 plasmid-encoded genes (*ehx*A, *esp*P, *etp*D, *kat*P and *tox*B), enterohemolysin (*ehx*A) and extracellular serine protease (*esp*P) were present in most, but not all EHEC and EPEC strains (Table 2). Conversely, toxin B gene (*tox*B), a homolog of EHEC factor for adherence gene (*efa*1), was found in only 2/6 (33.3%) human clinical EHEC and in only one bovine EHEC strain (2014-5-941B). The *efa*1 gene, not encoded on the pO157 plasmid, was present in a higher proportion of EHEC strains (41/49; 83.7%), compared to *tox*B; interestingly, bovine EPEC O103:H11 strain was also positive for *efa*1 gene (Table 3). All EPEC strains in this study were negative for the EAF plasmid.

**Table 3:** Putative virulence genes in enterohemorrhagic (EHEC), enteropathogenic (EPEC) and putative non-pathotype (*stx*/*eae* negative) *Escherichia coli* O103 strains of bovine and human origin^†^.

Protein and gene		Host origin, pathotype and serotype (no. of strains)						
		Bovine EHEC	Human EHEC		Bovine EPEC		Bovine putative non-pathotype	
		O103:H2	O103:H2	O103:H11	O103:H2	O103:H11	O103:H2	O103:H16
		(n = 43)	(n = 2)	(n = 4)	(n = 12)	(n = 1)	(n = 12)	(n = 1)
**EHEC factor for adherence**	***efa*1**	35	2	4	0	1	0	0
**Adherence protein**	***iha***	19	1	4	2	1	0	0
**EAST-1 heat-stable toxin**	***astA***	0	0	4	9	1	0	0
**SPATE**	***espI***	23	0	0	3	0	0	0
**Glutamic acid decarboxylase**	***gad***	43	2	4	12	1	12	1
**Increased serum survival**	***Iss***	43	2	4	9	1	12	1
**Long polar fimbriae**	***lpfA***	0	0	4	0	1	12	1
**Endonuclease colicin E2**	***celb***	20	0	0	0	1	0	0
**Colicin B**	***cba***	29	0	4	0	1	5	0
**Colicin M**	***cma***	1	0	0	0	0	5	0
**ABC transporter protein MchF**	***mchF***	0	0	0	0	0	5	0
**MchC protein**	***mchC***	0	0	0	0	0	5	0
**Microcin H47 part of colicin H**	***mchB***	0	0	0	0	0	5	0
**Microcin M part of colicin H**	***mcmA***	0	0	0	0	0	5	0

^†^Virulence genes were determined using Virulence Finder 1.4 [29].

The putative virulence genes that were present in the O103 strains are shown in Table 3. Of all adherence-based genes in EHEC and EPEC strains (Tables 2 and 3), only long polar fimbriae gene (*lpf*A) was present in putative non-pathotype strains. The *lpf*A gene was also present in all human EHEC O103:H11 strains (n = 4) and in the EPEC O103:H11 strain, but was not detected in O103:H2 strains within bovine and human EHEC and bovine EPEC subgroups or within any of the human EHEC control strains (O103:H2 12009, O157:H7 Sakai, O157:H7 EDL933). ABC transporter protein MchF (*mcf*F), MchC protein (*mch*C), Microcin H47 part of colicin H (*mch*B) and Microcin M part of colicin H (*mcm*A) genes were present in 5/12 (41.7%) bovine putative non-pathotype O103:H2 strains but absent in all other strains. The colicin M gene (*cma*) was found in 5 of 12 putative non-pathotype O103:H2 strains, but also in one bovine EHEC O103:H2 (strain 2014-5-1565C). Glutamic acid decarboxylase (*gad*) was present in all 75 strains. EAST-1 toxin gene (*ast*A), encoding for an enterotoxin, was in all O103:H11 strains (human EHEC and bovine EPEC) in the study, and in a majority of bovine EPEC O103:H2 strains (9/12), but not in any of the EHEC O103:H2 strains. Endonuclease colicin E2 gene (*celb*) was present in nearly half (20/43) of all bovine EHEC strains, and in the bovine EPEC O103:H11 strain, but absent from all other subgroups.

### Plasmid and prophage sequences

The complete plasmid replicon profiles of each genome are shown in tables S4, S5 and S6 Tables. Plasmid profiles exhibited some commonality among strains within an O103 subgroup but varied dramatically between subgroups. Plasmids from four incompatibility groups, including IncFIA(HI1), IncFII(pRSB107), IncFII(pSE11), IncX1 and IncY replicons were present at varying proportions in bovine EPEC O103:H12 strains, but absent from all other subgroups (Table 4). Similarly, strains from bovine EHEC were positive for IncA/C2, IncFII(pCoo) (enterotoxigenic *E*. *coli* associated plasmid), IncI2 and IncN plasmid replicons, while other subgroups were negative for these plasmids sequences. A high proportion of bovine EHEC (19/43; 44.2%) and the bovine EPEC O103:H11 strains were positive for Col156 plasmid sequence, while strains from all remaining subgroups were negative for this plasmid sequence. Among the nineteen total plasmid types identified in the strains, nearly half (9/19; 47.4%) belonged to the IncF incompatibility family. The IncFIB (*E*. *coli* K-12) plasmid sequence was most prevalent among the 75 strains, found in 39/43 (90.7%) bovine EHEC strains and in all human EHEC (6/6) and O103:H2 putative non-pathotype strains (12/12). The IncFIB plasmid sequence was present in the bovine EPEC O103:H11 strain, but absent from all EPEC O103:H2 strains.

**Table 4:** Number of enterohemorrhagic (EHEC), enteropathogenic (EPEC) and putative non-pathotype (*stx*/*eae* negative) *Escherichia coli* O103 strains of bovine and human origin positive for plasmids^†^.

Plasmid replicon	Host origin, pathotype and serotype (no. isolates tested)						
	Bovine EHEC	Human EHEC		Bovine EPEC		Bovine putative non-pathotype	
	O103:H2	O103:H2	O103:H11	O103:H2	O103:H11	O103:H2	O103:H16
	(n = 43)	(n = 2)	(n = 4)	(n = 12)	(n = 1)	(n = 12)	(n = 1)
**Col156**	19	0	0	0	1	0	0
**ColRNAI**	36	0	4	0	1	12	1
**IncA/C2**	3	0	0	0	0	0	0
**IncB/O/K/Z**	20	2	4	0	1	0	0
**IncFIA(HI1)**	0	0	0	2	0	0	0
**IncFIB(AP001918)**	39	2	4	0	1	12	0
**IncFIC(FII)**	0	0	0	0	0	12	0
**IncFII**	1	0	1	0	0	0	0
**IncFII(29)**	0	1	0	0	0	0	0
**IncFII(pCoo)**	3	0	0	0	0	0	0
**IncFII(pHN7A8)**	0	0	0	2	0	5	0
**IncFII(pRSB107)**	0	0	0	2	0	0	0
**IncFII(pSE11)**	0	0	0	9	0	0	0
**IncI2**	1	0	0	0	0	0	0
**IncN**	2	0	0	0	0	0	0
**IncP**	0	0	1	0	0	0	0
**IncX1**	0	0	0	2	0	0	0
**IncY**	0	0	0	7	0	0	0
**p0111**	2	0	2	0	1	0	0

^†^Plasmids were determined from whole genome sequences of strains using Plasmid Finder 1.3 [30].

The complete prophage profiles of each genome are shown in tables S7, S8 and S9 Tables. The 75 strains were positive for 20 different prophages (Table 5). Bovine EHEC strains were positive for the most number of these prophages (15/20), followed by bovine EPEC (11/20) and human EHEC strains (8/20). Bovine putative non-pathotype strains were positive for the fewest number of these prophages (5/20). A high proportion of bovine EHEC (28/43; 65.1%), human EHEC (5/6; 83.3%), and bovine EPEC (5/13; 38.5%) were positive for *Enterobacteria* phage P88, while only 7.7% (1/13) of bovine putative non-pathotype strains were positive for this prophage (Table 5). Interestingly, 61.5% of bovine putative non-pathotype strains (8/13) and 62.8% of bovine EHEC strains were positive for *Shigella* phage SfII, compared to none of the bovine EPEC strains and only 2 of 6 human EHEC strains.

**Table 5:** Number of enterohemorrhagic (EHEC), enteropathogenic (EPEC) and putative non-pathotype (*stx*/*eae* negative) *Escherichia coli* O103 strains of bovine and human origin positive for prophages^†^.

Prophage	Host origin, pathotype and serotype (no. isolates tested)						
	Bovine EHEC	Human EHEC		Bovine EPEC		Bovine putative non-pathotype	
	O103:H2	O103:H2	O103:H11	O103:H2	O103:H11	O103:H2	O103:H16
	(n = 43)	(n = 2)	(n = 4)	(n = 12)	(n = 1)	(n = 12)	(n = 1)
*Aeromonas* phage phiO18P	21	1	0	0	0	0	0
*Burkholderia* phage phiE255	0	0	0	0	0	5	0
*Enterobacteria* phage 933W	7	0	0	6	0	0	0
*Enterobacteria* phage BP-4795	8	0	0	2	0	0	0
*Enterobacteria* phage mEp043 c-1	8	1	0	0	0	0	0
*Enterobacteria* phage HK630	3	0	0	0	0	0	0
*Enterobacteria* phage lambda	19	2	0	4	0	0	0
*Enterobacteria* phage mEp460	3	0	0	8	0	0	0
*Enterobacteria* phage Mu	2	0	2	0	1	0	0
*Enterobacteria* phage P1	2	0	2	3	1	0	0
*Enterobacteria* phage P2	0	0	1	0	0	0	0
*Enterobacteria* phage P88	28	2	3	5	0	0	1
*Enterobacteria* phage Sf101	0	0	0	2	0	0	0
*Escherichia* phage D108	2	0	0	0	0	0	0
*Escherichia* phage TL-2011b	8	0	0	8	0	0	0
*Enterobacteria* phage Fels-2	0	0	0	0	0	7	0
*Salmonella* phage SEN34	0	0	0	0	0	3	0
*Shigella* phage SfII	27	2	0	0	0	7	1
*stx*2-converting phage 1717	5	0	0	1	0	0	0
*stx*2 converting phage vB_EcoP_24B	1	0	0	2	0	0	0

†Number of prophage sequences were determined from whole genome sequences of strains using Phage Search Tool Enhanced Release (PHASTER) [31, 32].

Only intact and questionable prophage counts based on PHASTER scores of >90 and 70–90, respectively, are shown.

### Phylogenetic relationships

**Fig 2:**
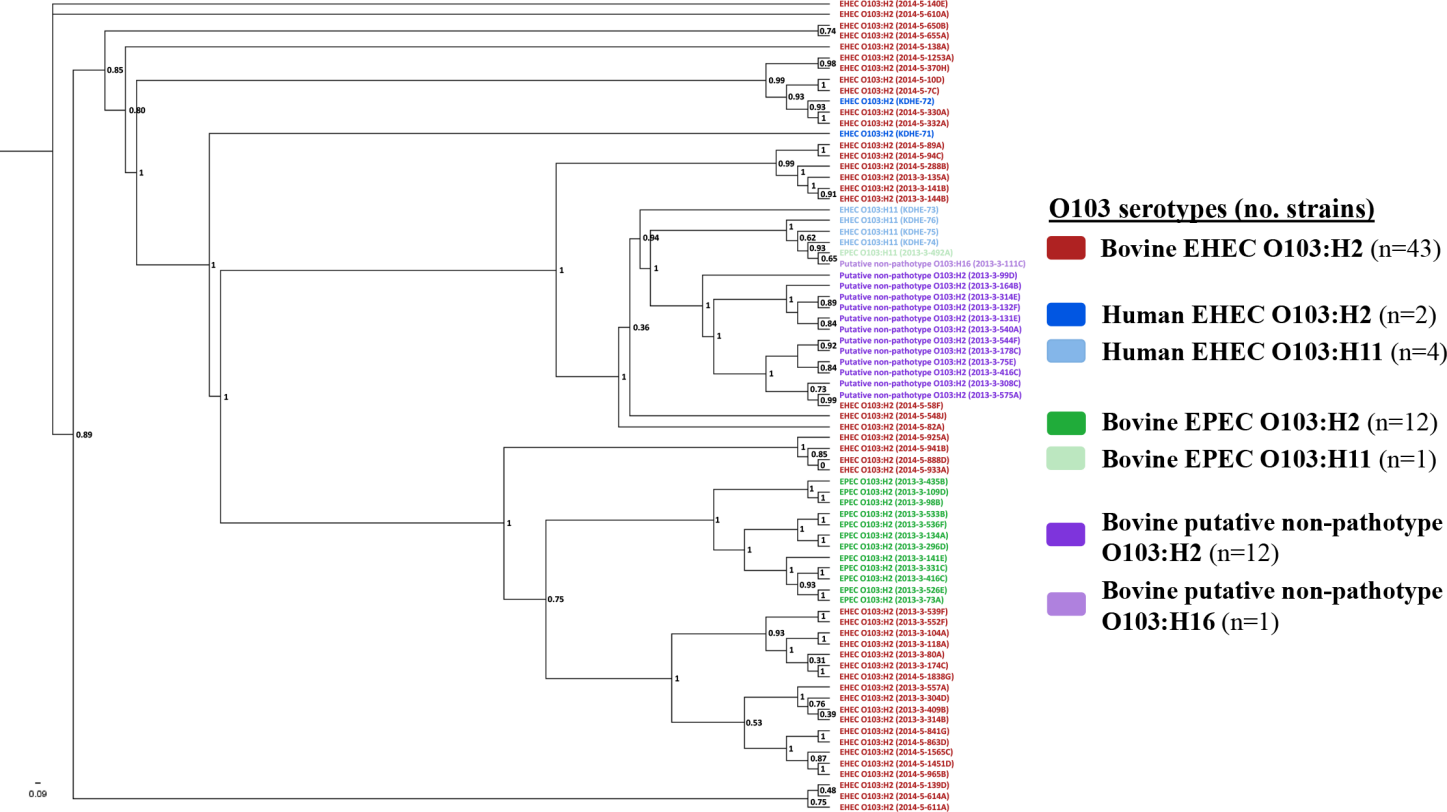
Proportional branch transformed phylogenetic tree^†^ of 75 strains of enterohemorrhagic (EHEC), enteropathogenic (EPEC) and putative non-pathotype (*stx*/*eae* negative) *Escherichia coli* O103 of bovine and human origin using FigTree 1.4. ^†^Numbers on the branches correspond to bootstrap values.

A maximum likelihood phylogenetic tree, based on core genome alignment of all 75 strains, was constructed using Parsnp v.1.2. The output file was proportional branch transformed using FigTree 1.4 (Fig 2). Overall, strains clustered according to pathotypes, with one notable exception: bovine EPEC O103:H11 strain (2013-3-492A) was more closely related to a human EHEC O103:H11 (strain KSU-74) than to any of the other bovine EPEC strains included in the study (Fig 2). All EPEC O103:H2 strains clustered together and putative non-pathotype strains exhibited a similar clustering. One human EHEC O103:H2 strain (KSU-72) was more closely related to two bovine EHEC O103:H2 strains (2014-5-330A and 2014-5-332A) than to the other human EHEC O103:H2 strain (KSU-71) included in the study.

**Fig 3:**
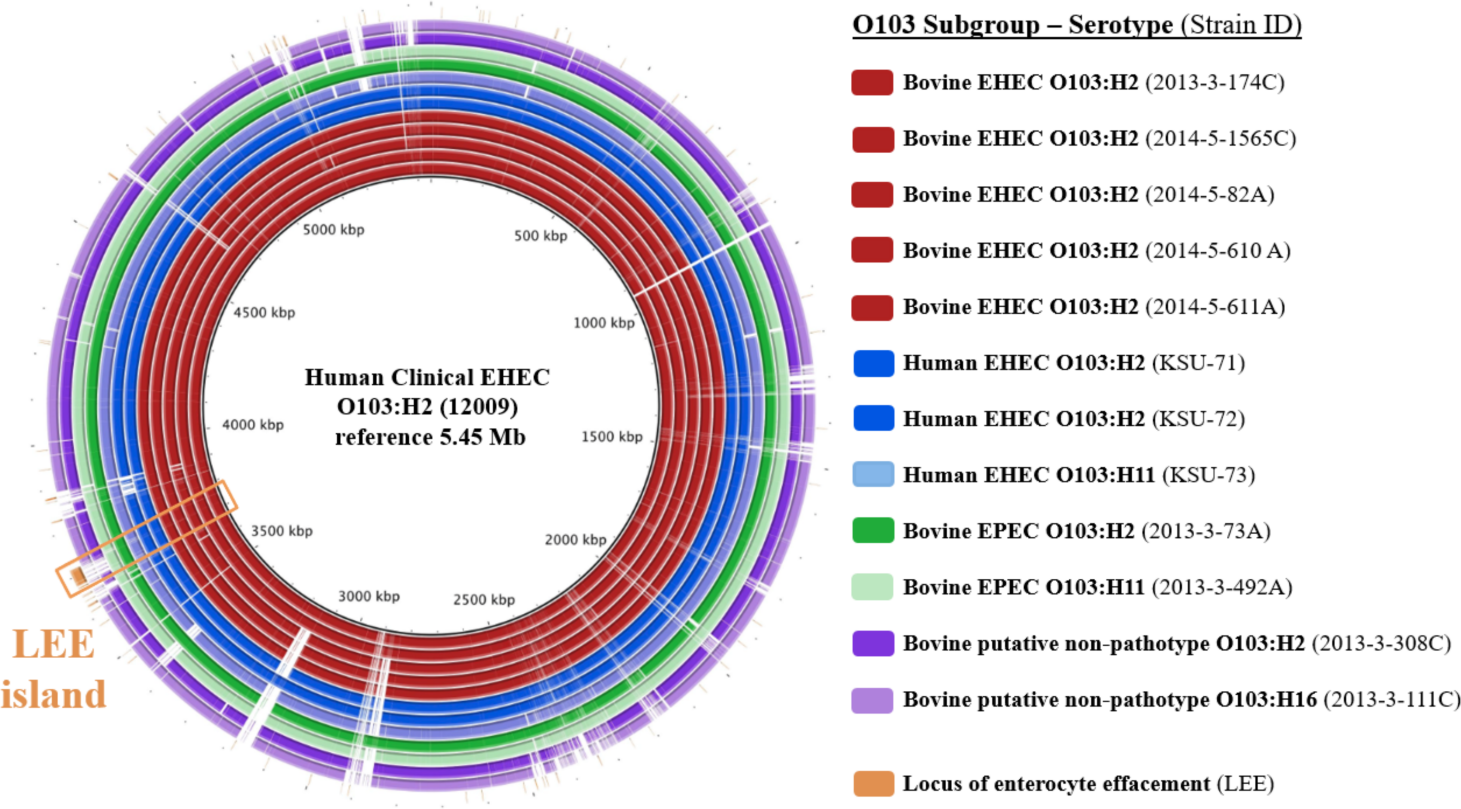
Multiple genome comparison of representative strains of enterohemorrhagic (EHEC), enteropathogenic (EPEC) and putative non-pathotype (*stx*/*eae* negative) *Escherichia coli* O103 strains of bovine and human origin using BLAST Ring Image Generator (BRIG) v0.95. ^†^The nucleotide sequence (45,325 bp) of the locus of enterocyte effacement (LEE) pathogenicity island (GenBank accession no.: AF071034.1) was mapped for comparison of LEE between target strains.

Based on clustering patterns in Fig 2, representative strains were selected from observed serotypes (O103:H2, O103:H11, and O103:H16) within each O103 subgroup (bovine EHEC, human EHEC, bovine EPEC, and bovine putative non-pathotype) as input for BLAST Ring Image Generator (BRIG) v0.95 [37]. The draft genomes of these target strains are represented by the concentric rings in the BRIG plot; any missing portions of these rings represent nucleotide sequences missing from the target strains in comparison to a central reference strain (EHEC O103:H2 strain 12009; Fig 3). Putative non-pathotype strains (2013-3-308C and 2013-3-111C) displayed the largest degree of sequence divergence to the reference strain. As expected, the LEE island (45,325 bp), which encodes for the *eae* gene and other Type III secretion effectors, was present in all EHEC and EPEC strains, but absent in the putative non-pathotype strains. Interestingly, a relatively large unknown sequence (~40,000 bp) from the reference strain was present in 2/5 bovine EHEC O103:H2 strains (2013-3-174C, 2014-5-1565C) and in 1/3 human EHEC strains (KSU-72), but absent in all other EHEC, EPEC, and putative non-pathotype strains. It is worth noting that the three strains positive for the unknown sequence were not positive for any virulence genes not found in the remaining strains tested. Strains 2013-3-174C and 2014-5-1565C of bovine EHEC O103:H2 had higher sequence similarity with the human clinical O103:H2 reference strain than to any of the human clinical EHEC target strains.

## Discussion

*Escherichia coli* O103 is the third most common STEC (next to O157 and O26) implicated in human STEC infections [1, 38]. Based on our studies, serogroup O103 is the second most prevalent STEC (next to O157) shed in cattle feces [12, 21]. Brooks *et al*. [38] have reported that 117 human clinical O103 isolates, submitted to CDC from 1983 to 2002, were positive for *stx*1 and negative for *stx*2, and included only four flagellar types, H2, H11, H25 and non-motile. Similarly, all Shiga toxin-producing strains of cattle origin in this study (n = 43) were positive for *stx*1 gene only, however, all possessed the H2 flagellar type. The predominance of the H2 flagellar type in bovine strains is in agreement with previous reports of O103 strains in cattle and sheep [20, 39–41]. The majority of EHEC strains (48/49; 98.0%) in our study had Shiga toxin 1a (*stx*1a) gene. Söderlund *et al*. [20] report Shiga toxin 1a (*stx*1a) subtype present in five EHEC O103:H2 isolated from Swedish cattle. Similar to findings from previous studies [20, 33], all EHEC/EPEC O103:H2 and O103:H11 strains carried epsilon and beta1 *eae* subtypes, respectively. All EPEC strains included in this study were considered atypical, as indicated by the absence of the EAF plasmid, a finding also in agreement with previous studies [20, 42, 43].

All EHEC O103 strains in this study (43 bovine and 6 human strains) had a higher number of genes on mobile elements (prophages, transposable elements, and plasmids) compared to the bovine EPEC (except for one O103:H11 strain) and putative non-pathotype strains. Significant differences in the genome size observed among the O103 subgroups are reflective of the number of genes from mobile elements. However, one bovine EPEC O103:H11 strain (2013-3-492A) was an exception as its genome size and number of genes on mobile elements were more comparable to EHEC strains (Fig 1); furthermore, this strain was more closely related to a human EHEC O103:H11 strain (KSU-74) than to any of the EPEC strains (Fig 2). Also, the virulence gene profile of the EPEC O103:H11 strain 2013-3-492A more closely resembled the virulence gene profiles of the EHEC O103 subgroup than that of the bovine EPEC O103 subgroup. Furthermore, the strain is positive for *stx*1 bacteriophage insertion site (*yehV*) and bacteriophage-*yehV* right and left junctions [44], suggesting that the EPEC O103:H11 strain may be capable of acquiring and/or had once acquired but lost *stx* gene(s). This suggests that much of the genetic diversity in *E*. *coli* O103 strains shed in cattle feces can be attributed to the loss or to acquisition of mobile genetic elements [45].

Similar to the phylogenetic clustering of bovine EHEC and EPEC O103:H2 strains reported in Söderlund *et al*. [20], strains in this study largely clustered by pathotype (Fig 2). A genome-wide visual comparison between representative strains from observed serotypes (O103:H2, O103:H11, O103:H16) within each O103 subgroup (bovine EHEC, human EHEC, bovine EPEC, and bovine putative non-pathotype) showed clear differences in the sequence identity between target strains (Fig 3). Interestingly, two of the bovine EHEC O103:H2 strains (2013-3-174C and 2014-5-1565C) shared more sequence identity with the clinical reference strain than did the human EHEC strains included in Fig 3, which may be an indication of the virulence potential of these strains. It is clear that the EHEC and EPEC strains have acquired more genetic elements during the course of their evolution in comparison to the putative non-pathotype strains. Although overall number of genes implicated in virulence, disease and defense was comparable among all 69 bovine strains, a closer examination revealed key differences in virulence gene profiles of O103 subgroups and serotypes within subgroups.

### LEE effector genes

The chromosomal LEE pathogenicity island carries genes that encode for intimin (*eae*), translocated intimin receptor protein (*tir*), and type III secretion system effector proteins (*esp*A and *esp*B). Studies have shown that without any one of these genes (*eae*, *tir*, *esp*A, *esp*B), attaching and effacing (A/E) *E*. *coli* are unable to produce their characteristic A/E lesions [46–48]. The *esp*F gene is also LEE encoded, but unlike the other LEE genes that were present in all EHEC and EPEC strains, a small number of bovine EPEC (3/13) and EHEC (4/43) strains were *esp*F-negative. Although *esp*F contributes to the disruption of intestinal barrier function during attachment, McNamara *et al*. [49] have shown that the gene is not required for A/E lesion formation. Other type III effector genes (*cif*, *esp*J, and *tcc*P) were variably present in the EHEC and EPEC strains, possibly, because they are prophage-encoded genes. Although *cif* and *esp*J genes enhance attachment, *in vivo* and/or *in vitro* studies have shown that A/E lesions are not significantly inhibited in the absence of either gene [50, 51]. Garmendia *et al*. [52] have shown that *tir*-cytoskeleton coupling protein gene (*tcc*P) assists in the translocation of the intimin receptor protein during bacterial attachment. In the same study, *tcc*P mutants were unable to trigger A/E lesions on *in vitro-*inoculated HeLa epithelial cells. Considering its seemingly critical importance in type III secretory system-related disease outcomes, it is surprising that not all human clinical EHEC were positive for the *tcc*P gene. Garmendia *et al*. [52] reported that *tir* translocation was not affected in *tcc*P mutants, therefore, it is possible that bacterial attachment and expression of other virulence factors in *tcc*P-negative EHEC could contribute to A/E lesions.

### Non-LEE effector genes

Non-LEE effector (*nle*) genes, including *nle*A, *nle*B and *nle*C, have been associated with HUS-causing strains of EHEC [53] and were present in varying proportions within EHEC and EPEC O103 subgroups in this study. In two independent studies, Δ*nle*A [54] and Δ*nle*B mutant strains of *Citrobacter rodentium* [55] were unable to cause mortality in inoculated mice. Wickham *et al*. [55] also reported a three-log decrease (10^6^ vs. 10^3^) in infectious dose for *nle*B wildtype- compared to Δ*nle*B-mutant, which highlights the importance of *nle*B gene as it relates to the low infectious dose of EHEC strains. The *nle*C gene serves to down-regulate host NF-ĸB signaling pathway in efforts to disrupt immune clearance of invading bacteria [56]. Although *nle*C has also been significantly associated with HUS-causing strains [53], it was present only in 4 of 6 human clinical EHEC strains, but in 53.5% (23/43) of bovine EHEC strains.

### pO157 plasmid encoded virulence genes

The pO157 plasmid (~93 kb) carries a number of virulence genes implicated in EHEC virulence [57] and is present in nearly all clinical O157:H7 strains [58]. Major pO157 plasmid-encoded genes, *ehx*A, *esp*P, *etp*D, *kat*P and *tox*B, were present in many EHEC and EPEC O103 strains. The enterohemolysin gene (*ehx*A), present in all EHEC (49/49) and nearly all EPEC (12/13) strains in this study, encodes for a pore-forming toxin, which elicits *in vivo* production of IL-1β from human mononuclear cells, a commonly expressed cytokine during HUS infections [59]. The extracellular serine protease gene (*esp*P) was found in almost all EHEC and EPEC strains and is considered to contribute to hemorrhagic colitis via the cleavage of pepsin A and human coagulation factor V [60].

The *etp*D, *kat*P and *tox*B genes, located on the pO157 plasmid, were less frequently present in EHEC and EPEC strains, compared to *ehx*A and *esp*P genes. Schmidt *et al*. [61] report that EHEC type II secretion pathway (*etp*) genes are not commonly detected (~10%) in bovine EHEC isolated from feces. In this study, *etp*D gene was present in 9 of 43 (20.9%) of bovine EHEC strains, but absent in the other subgroups. Brunder *et al*. [62] report a close association between the presence of *ehx*A and the catalase peroxidase gene (*kat*P) in EHEC O157:H7 strains. We observed a similar trend for bovine and human EHEC; however, *ehx*A was present in a majority (11/12) of bovine EPEC O103:H2, whereas *kat*P was absent in all of those strains. The *tox*B gene, identified by Tatsuno *et al*. [63], is a homolog of EHEC factor for adherence gene (*efa*1), carried on the pO157 plasmid and is commonly present in clinical EHEC O157:H7. In a study examining the prevalence of *tox*B in O157 and major non-O157 EHEC and EPEC of clinical and animal origin, Tozzoli *et al*. [64] report all O103 strains used in their study were negative for the gene. In the current study, 3 of 6 human EHEC strains were positive for *tox*B. Yet, the gene was present in only 1/43 bovine EHEC strains and in the single bovine EPEC O103:H11 strain. Although *tox*B is not required for formation of A/E lesions, Tatsuno *et al*. [63] showed that expression of *tox*B does lead to enhanced virulence by increasing expression of major LEE-encoded effector genes including *esp*A, *esp*B and *tir*.

### Other virulence genes

Interestingly, *lpf*A was the only adherence-based virulence gene present in the bovine putative non-pathotype O103:H2 strains (n = 12), yet the gene was absent in all EHEC (n = 43) and EPEC O103:H2 (n = 12) strains, suggesting possible loss of *lpf*A gene by O103:H2 serotype at some point during the course of acquiring new genetic elements. The gene for increased serum survival (*iss*) was prevalent in all 75 strains. The *iss* gene is often associated with avian pathogenic *E*. *coli* (APEC) that cause colibacillosis in poultry, and serves as a genetic marker for APEC strains [4]. Among APEC, the *iss* gene is carried by a ColV plasmid [65] that in addition to conferring increased virulence and fitness traits, also encodes for multidrug resistance [66].

The *E*. *coli* secreted protease island encoded gene (*esp*I) is considered part of the family of extracellular proteases known as SPATE, or serine protease autotransporters of *Enterobacteriaceae* [67]. The *esp*I gene is harbored on the O91:H¯ pathogenicity island and previously reported to occur exclusively in a LEE-negative subgroup of STEC that carry a *stx*2d gene variant [68]. Krüger *et al*. [69] also report detection of *esp*I gene exclusively in *stx*2- (but not *stx*1) positive *E*. *coli* O26:H11 strains of clinical, bovine and food origin. In our study, *esp*I gene was present in more than half (23/43; 53.5%) of all bovine EHEC O103:H2 that were *stx*1a positive; *esp*I gene was also present in three of 12 bovine EPEC O103:H2 strains. These results are in contrast with previous studies linking the *esp*I gene to *stx*2-carrying EHEC only [68, 69] and may be the first time *esp*I gene has been reported in bovine EHEC and EPEC O103 strains.

### Plasmid and prophage sequences

Some of these plasmid sequences are originally associated with non-*E*. *coli* bacteria, including *Klebsiella pneumoniae* (ColRNAI and IncA/C2), *Salmonella typhi* (IncFIA(HI1)), *Salmonella typhimurium* (IncN) and *Pseudomonas aeruginosa* (IncP), which further highlights the mobility of these genetic elements. Many of the plasmids, including IncA/C2, IncFII, IncFII(pHN7A8), IncFII(pRSB107), IncN and IncX1 have also been associated with antimicrobial resistance determinants and/or other putative virulence-associated functions, that in some cases have been the causative genetic element behind human outbreaks [70]. The IncF incompatibility family represents the majority of virulence-associated plasmids carried by *E*. *coli* [71], therefore it may not be surprising that IncF plasmids represented nearly half (96/218; 44.0%) of all total plasmids identified in the strains used in this study.

Similarly, non-*E*. *coli* prophage sequences, including *Aeromonas* phage phiO18P, *Burkholderia* phage phiE255, *Salmonella* phage SEN34 and *Shigella* phage SfII, were found in many of the strains, which further demonstrates the mobility of these genetic elements. The most and least prophage diversity, defined by total number of different prophages carried by an O103 subgroup, was found in bovine EHEC and bovine putative non-pathotype strains, respectively, which also highlights the differences in mobile content found between these subgroups.

## Conclusion

The virulence gene profiles of the bovine and human EHEC, bovine (atypical) EPEC and putative non-pathotype strains of *E*. *coli* O103 were quite diverse. The difference in the number of strains tested within each subgroup and lack of publicly available O103 genome sequences may have limited the strength of comparison. Although the *in silico* analysis performed here does not provide phenotypic evidence of virulence contributions, a number of major and putative virulence genes were comparable among bovine and human EHEC O103 strains, which may indicate the potential for bovine EHEC O103 to cause human infection. The bovine EPEC O103:H11 strain also shared similar virulence gene and plasmid profiles with human EHEC O103:H11 strains, raising the possibility that the EPEC may have lost its *stx* prophage. Regardless, the *in silico* data highlight the numerous virulence genes carried on mobile genetic elements (prophages, transposable elements, and plasmids) that contribute to the plasticity of bovine EHEC or EPEC. Genome size and number of genes from mobile elements were strongly correlated among the O103 subgroups. The putative non-pathotype strains had the smallest genome size and carried the fewest overall number of mobile genes and perhaps related to this, lacked any specific major or putative mobile virulence genes. The EPEC strains in this study had larger genomes and were positive for a higher number of specific virulence genes compared to putative non-pathotype strains. Excluding the outlying EPEC O103:H11 strain, the EHEC overshadowed EPEC, and putative non-pathotype subgroups in both these categories, which raises the question whether progenitor EHEC bacteria are more genetically predisposed toward acquiring certain mobile elements that could confer virulence. Conversely, putative virulence genes that allow for increased EHEC survival within the environment or within a host may afford EHEC with increased opportunity to acquire mobile genetic elements. We believe that the diversity of pathotypes of *E*. *coli* O103 harbored and shed in the feces of cattle is reflective of the loss or acquisition of genes carried on mobile genetic elements. The environmental and biological mechanisms that allow for loss or acquisition of virulence genes by EHEC and EPEC and putative non-pathotype strains remain an exciting frontier for the whole-genome sequence-based analysis of *E*. *coli* pathotypes.

## Supporting information

S1 Table Virulence gene profiles^†^ of enterohemorrhagic *Escherichia coli* (EHEC) O103:H2 strains isolated from cattle feces collected from nine feedlots in the Midwest. ^†^Virulence genes were determined using Virulence Finder 1.4 [29].
(DOCX)

S2 Table Virulence gene profiles^†^ of enteropathogenic *Escherichia coli* (EPEC) O103 and *E*. *coli* O103 strains negative for Shiga toxin and intimin genes (O-group) isolated from cattle feces collected from a Midwest feedlot. ^†^Virulence genes were determined using Virulence Finder 1.4 [29].
(DOCX)

S3 Table Virulence gene profiles^†^ of clinical human enterohemorrhagic *Escherichia coli* (EHEC) O103 strains. ^†^Virulence genes were determined using Virulence Finder 1.4 [29].
^‡^Control strains were included for comparison and result from the testing of genomic and plasmid (O103:H2 12009, NC_013354.1; Sakai, NC_002128.1 and NC_002127.1; EDL933, AF074613.1) DNA sequences available at GenBank.
(DOCX)

S4 Table Plasmid profiles^†^ of enterohemorrhagic *Escherichia coli* (EHEC) O103:H2 strains isolated from cattle feces collected from nine feedlots in the Midwest. ^†^Plasmids were determined from whole genome sequences of strains using Plasmid Finder 1.3 [30].
(DOCX)

S5 Table Plasmid profiles^†^ of enteropathogenic *Escherichia coli* (EPEC) O103 and *E*. *coli* O103 strains negative for Shiga toxin and intimin genes (O-group) isolated from cattle feces collected from a Midwest feedlot. ^†^Plasmids were determined from whole genome sequences of strains using Plasmid Finder 1.3 [30].
(DOCX)

S6 Table Plasmid profiles^†^ of clinical human enterohemorrhagic *Escherichia coli* (EHEC) O103 strains. †Plasmids were determined from whole genome sequences of strains using Plasmid Finder 1.3 [30].
*Control strains were included for comparison and result from the testing of genomic and plasmid (O103:H2 12009, NC_013354.1; Sakai, NC_002128.1 and NC_002127.1; EDL933, AF074613.1) DNA sequences available at GenBank.
(DOCX)

S7 Table Prophage profiles^†^ of enterohemorrhagic *Escherichia coli* (EHEC) O103:H2 strains isolated from cattle feces collected from nine feedlots in the Midwest. ^†^Prophage sequences were determined from whole genome sequences of strains using Phage Search Tool Enhanced Release (PHASTER) [31, 32]. Only intact and questionable prophage counts based on PHASTER scores of >90 and 70–90, respectively, are shown.
(DOCX)

S8 Table Prophage profiles^†^ of enteropathogenic *Escherichia coli* (EPEC) O103 and *E*. *coli* O103 strains negative for Shiga toxin and intimin genes (O-group) isolated from cattle feces collected from a Midwest feedlot. ^†^Prophage sequences were determined from whole genome sequences of strains using Phage Search Tool Enhanced Release (PHASTER) [31, 32]. Only intact and questionable prophage counts based on PHASTER scores of >90 and 70–90, respectively, are shown.
(DOCX)

S9 Table Prophage profiles^†^ of clinical human enterohemorrhagic *Escherichia coli* (EHEC) O103 strains. ^†^Prophage sequences were determined from whole genome sequences of strains using Phage Search Tool Enhanced Release (PHASTER) [31, 32]. Only intact and questionable prophage counts based on PHASTER scores of >90 and 70–90, respectively, are shown.
*Control strains were included for comparison and result from the testing of genomic and plasmid (O103:H2 12009, NC_013354.1; Sakai, NC_002128.1 and NC_002127.1; EDL933, AF074613.1) DNA sequences available at GenBank.
(DOCX)
