# Supplementary material for: Comparative genomics reveals differences in mobile virulence genes of Escherichia coli O103 pathotypes of bovine fecal origin
Source: PLoS One. 2018 Feb 1;13(2):e0191362. doi: 10.1371/journal.pone.0191362 (PMC5794082; doi:10.1371/journal.pone.0191362)
Supplement: S1 Table — †Virulence genes were determined using Virulence Finder 1.4 [29]. (DOCX) [file pone.0191362.s001.docx]

**S1 Table: Virulence gene profiles^†^ of enterohemorrhagic *Escherichia coli* (EHEC) O103:H2 strains isolated from cattle feces collected from nine feedlots in the Midwest.**


^†^Virulence genes were determined using Virulence Finder 1.4 [29].

**Supplemental Table 2: Virulence gene profiles^†^ of enteropathogenic *Escherichia coli* (EPEC) O103 and *E. coli* O103 strains negative for Shiga toxin and intimin genes (O-group) isolated from cattle feces collected from a Midwest feedlot.**

^†^Virulence genes were determined using Virulence Finder 1.4 [29].

**Supplemental Table 3: Virulence gene profiles^†^ of clinical human enterohemorrhagic *Escherichia coli* (EHEC) O103 strains**

^†^Virulence genes were determined using Virulence Finder 1.4 [29].
^‡^Control strains were included for comparison and result from the testing of genomic and plasmid (O103:H2 12009, NC_013354.1; Sakai, NC_002128.1 and NC_002127.1; EDL933, AF074613.1) DNA sequences available at GenBank.

**Supplemental Table 4: Plasmid profiles^†^ of enterohemorrhagic *Escherichia coli* (EHEC) O103:H2 strains isolated from cattle feces collected from nine feedlots in the Midwest.**


^†^Plasmids were determined from whole genome sequences of strains using Plasmid Finder 1.3 [30].

**Supplemental Table 5: Plasmid profiles^†^ of enteropathogenic *Escherichia coli* (EPEC) O103 and *E. coli* O103 strains negative for Shiga toxin and intimin genes (O-group) isolated from cattle feces collected from a Midwest feedlot.**

^†^Plasmids were determined from whole genome sequences of strains using Plasmid Finder 1.3 [30].

**Supplemental Table 6: Plasmid profiles^†^ of clinical human enterohemorrhagic *Escherichia coli* (EHEC) O103 strains**


^†^Plasmids were determined from whole genome sequences of strains using Plasmid Finder 1.3 [30]

^*^Control strains were included for comparison and result from the testing of genomic and plasmid (O103:H2 12009, NC_013354.1; Sakai, NC_002128.1 and NC_002127.1; EDL933, AF074613.1) DNA sequences available at GenBank.

**Supplemental Table 7: Prophage profiles^†^ of enterohemorrhagic *Escherichia coli* (EHEC) O103:H2 strains isolated from cattle feces collected from nine feedlots in the Midwest.**

**^†^**Prophage sequences were determined from whole genome sequences of strains using Phage Search Tool Enhanced Release (PHASTER) [31, 32]. Only intact and questionable prophage counts based on PHASTER scores of >90 and 70-90, respectively, are shown.

**Supplemental Table 8: Prophage profiles^†^ of enteropathogenic *Escherichia coli* (EPEC) O103 and *E. coli* O103 strains negative for Shiga toxin and intimin genes (O-group) isolated from cattle feces collected from a Midwest feedlot.**

**^†^**Prophage sequences were determined from whole genome sequences of strains using Phage Search Tool Enhanced Release (PHASTER) [31, 32]. Only intact and questionable prophage counts based on PHASTER scores of >90 and 70-90, respectively, are shown.

**Supplemental Table 9: Prophage profiles^†^ of clinical human enterohemorrhagic *Escherichia coli* (EHEC) O103 strains**

**^†^**Prophage sequences were determined from whole genome sequences of strains using Phage Search Tool Enhanced Release (PHASTER) [31, 32]. Only intact and questionable prophage counts based on PHASTER scores of >90 and 70-90, respectively, are shown.

^*^Control strains were included for comparison and result from the testing of genomic and plasmid (O103:H2 12009, NC_013354.1; Sakai, NC_002128.1 and NC_002127.1; EDL933, AF074613.1) DNA sequences available at GenBank.
