# Supplementary material for: Comparative genomics reveals differences in mobile virulence genes of Escherichia coli O103 pathotypes of bovine fecal origin
Source: PLoS One. 2018 Feb 1;13(2):e0191362. doi: 10.1371/journal.pone.0191362 (PMC5794082; doi:10.1371/journal.pone.0191362)
Supplement: S2 Table — †Virulence genes were determined using Virulence Finder 1.4 [29]. (DOCX) [file pone.0191362.s002.docx]

**S2 Table: Virulence gene profiles^†^ of enteropathogenic *Escherichia coli* (EPEC) O103 and *E. coli* O103 strains negative for Shiga toxin and intimin genes (O-group) isolated from cattle feces collected from a Midwest feedlot.**

^†^Virulence genes were determined using Virulence Finder 1.4 [29].
