# Supplementary material for: Comparative genomics reveals differences in mobile virulence genes of Escherichia coli O103 pathotypes of bovine fecal origin
Source: PLoS One. 2018 Feb 1;13(2):e0191362. doi: 10.1371/journal.pone.0191362 (PMC5794082; doi:10.1371/journal.pone.0191362)
Supplement: S3 Table — †Virulence genes were determined using Virulence Finder 1.4 [29]. ‡Control strains were included for comparison and result from the testing of genomic and plasmid (O103:H2 12009, NC_013354.1; Sakai, NC_002128.1 and NC_002127.1; EDL933, AF074613.1) DNA sequences available at GenBank. (DOCX) [file pone.0191362.s003.docx]

**S3 Table: Virulence gene profiles^†^ of clinical human enterohemorrhagic *Escherichia coli* (EHEC) O103 strains**

^†^Virulence genes were determined using Virulence Finder 1.4 [29].
^‡^Control strains were included for comparison and result from the testing of genomic and plasmid (O103:H2 12009, NC_013354.1; Sakai, NC_002128.1 and NC_002127.1; EDL933, AF074613.1) DNA sequences available at GenBank.
