# Supplementary material for: Comparative genomics reveals differences in mobile virulence genes of Escherichia coli O103 pathotypes of bovine fecal origin
Source: PLoS One. 2018 Feb 1;13(2):e0191362. doi: 10.1371/journal.pone.0191362 (PMC5794082; doi:10.1371/journal.pone.0191362)
Supplement: S4 Table — †Plasmids were determined from whole genome sequences of strains using Plasmid Finder 1.3 [30]. (DOCX) [file pone.0191362.s004.docx]

**S4 Table: Plasmid profiles^†^ of enterohemorrhagic *Escherichia coli* (EHEC) O103:H2 strains isolated from cattle feces collected from nine feedlots in the Midwest.**


^†^Plasmids were determined from whole genome sequences of strains using Plasmid Finder 1.3 [30].
