# Supplementary material for: Comparative genomics reveals differences in mobile virulence genes of Escherichia coli O103 pathotypes of bovine fecal origin
Source: PLoS One. 2018 Feb 1;13(2):e0191362. doi: 10.1371/journal.pone.0191362 (PMC5794082; doi:10.1371/journal.pone.0191362)
Supplement: S7 Table — †Prophage sequences were determined from whole genome sequences of strains using Phage Search Tool Enhanced Release (PHASTER) [31, 32]. Only intact and questionable prophage counts based on PHASTER scores of >90 and 70–90, respectively, are shown. (DOCX) [file pone.0191362.s007.docx]

**S7 Table: Prophage profiles^†^ of enterohemorrhagic *Escherichia coli* (EHEC) O103:H2 strains isolated from cattle feces collected from nine feedlots in the Midwest.**

**^†^**Prophage sequences were determined from whole genome sequences of strains using Phage Search Tool Enhanced Release (PHASTER) [31, 32]. Only intact and questionable prophage counts based on PHASTER scores of >90 and 70-90, respectively, are shown.
