# Supplementary material for: Comparative genomics reveals differences in mobile virulence genes of Escherichia coli O103 pathotypes of bovine fecal origin
Source: PLoS One. 2018 Feb 1;13(2):e0191362. doi: 10.1371/journal.pone.0191362 (PMC5794082; doi:10.1371/journal.pone.0191362)
Supplement: S8 Table — †Prophage sequences were determined from whole genome sequences of strains using Phage Search Tool Enhanced Release (PHASTER) [31, 32]. Only intact and questionable prophage counts based on PHASTER scores of >90 and 70–90, respectively, are shown. (DOCX) [file pone.0191362.s008.docx]

**S8 Table: Prophage profiles^†^ of enteropathogenic *Escherichia coli* (EPEC) O103 and *E. coli* O103 strains negative for Shiga toxin and intimin genes (O-group) isolated from cattle feces collected from a Midwest feedlot.**

**^†^**Prophage sequences were determined from whole genome sequences of strains using Phage Search Tool Enhanced Release (PHASTER) [31, 32]. Only intact and questionable prophage counts based on PHASTER scores of >90 and 70-90, respectively, are shown.
